# Marine accident learning with fuzzy cognitive maps (MALFCMs)

**DOI:** 10.1016/j.mex.2020.100940

**Published:** 2020-05-28

**Authors:** Beatriz Navas de Maya, Rafet Emek Kurt

**Affiliations:** University of Strathclyde, 100 Montrose St, Glasgow City (GLG), Glasgow G4 0LZ, United Kingdom

**Keywords:** Human factors, Risk factors, Accident prevention, Accident investigation, Shipping accidents, Maritime safety

## Abstract

Statistical analysis of past accidents in maritime may demonstrate the trends for certain contributing factors in accidents, however, there is a lack of a suitable technique to model the complex interrelations between these factors. Due to aforementioned complex interrelations and insufficient information stored in accident databases, it was not possible to understand the importance of each factor in accidents, which prevented researchers from considering these factors in risk assessments. Therefore, there is a need for a capable technique to estimate the importance of each factor. The results of such a technique can be used to inform risk assessments and predict the effectiveness of risk control options. Thus, this study introduces a new technique for Marine Accident Learning with Fuzzy Cognitive Maps (MALFCMs). The novelty of MALFCM is the application of fuzzy cognitive maps (FCMs) to model the relationships of maritime accident contributors by directly learning from an accident database as well as having the ability to combine expert opinion. As each fuzzy cognitive map is derived from real occurrences supported by expert opinion, the results can be considered more objective. Thus, MALFCM may overcome the main disadvantage of fuzzy cognitive maps by eliminating or controlling the subjectivity in results.

• A novel MALFCM method to weight human-contributing factors into maritime accidents has been developed.
• With MALFCM method the main disadvantage of traditional FCMs is overcome.
• The MALFCM method can produce logical results even by solely using information from historical data in the absence of expert judgement.

## Highlights

• A novel MALFCM method to weight human-contributing factors into maritime accidents has been developed.
• With MALFCM method the main disadvantage of traditional FCMs is overcome.
• The MALFCM method can produce logical results even by solely using information from historical data in the absence of expert judgement.

## Graphical abstract

**Figure:**
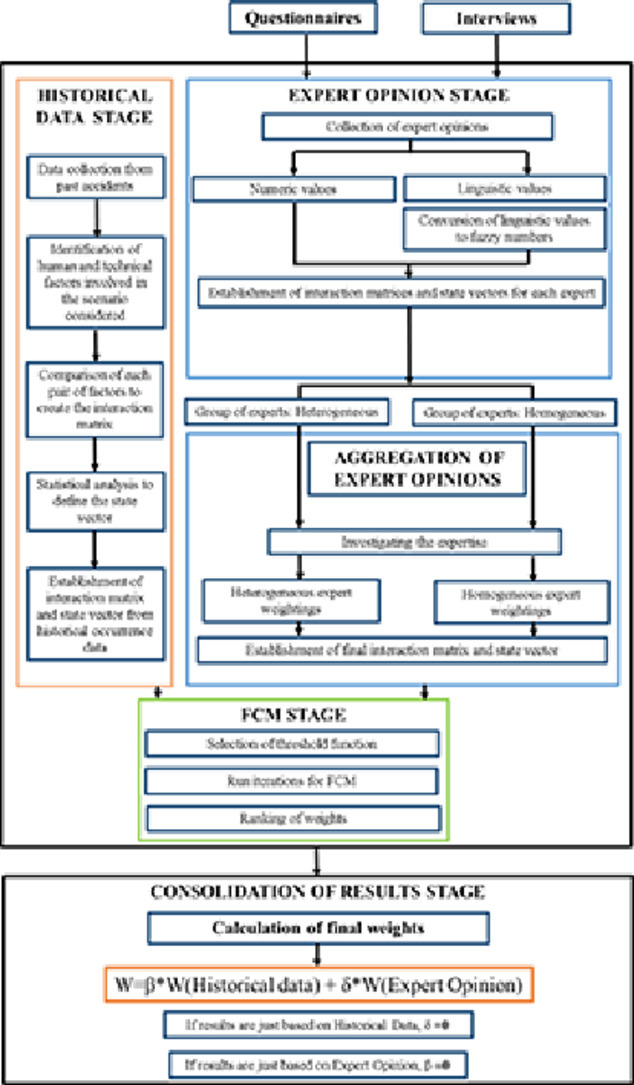

Specifications table

**Table:** 

Subject Area	Engineering
More specific subject area	*Accident investigation*
Method name	*Marine Accident Learning with Fuzzy Cognitive Maps (MALFCMs)*
Name and reference of original method	*Kosko, B.*[2]*. "Fuzzy cognitive maps." International journal of man-machine studies 24(1): 65–75.*
	*According with Kosko, Fuzzy cognitive maps (FCMs) are fuzzy-graph structures for representing causal reasoning. Their fuzziness allows hazy degrees of causality between hazy causal objects (concepts). Their graph structure allows systematic causal propagation, in particular forward and backward chaining, and it allows knowledge bases to be grown by connecting different FCMs*
**Resource availability**	*The method relies on having access to a historical accident database*

## Method details

Although FCM is an alternative and powerful method to model and analyze dynamic interactions between concepts or systems, it has an important limitation. As FCMs are designed to transcribe experts’ opinion, its weaknesses lay on the uncertainty related with each expert's response. As a result, an FCM can equally encode the experts’ lack of knowledge. Therefore, the reliability of a traditional FCM is linked to the experts’ knowledge, background and familiarity with the topic that is being addressed. In order to overcome this limitation, a method for Marine Accident Learning with Fuzzy Cognitive Maps (MALFCMs), which differs from the traditional FCM approach, is proposed in this paper. Within this new method, each MALFCM will be developed as follows:

• First, an historical accident database will be analyzed, in order to identify relationships amongst accident contributing factors from past accident experiences. Thus, factors’ weightings will be obtained by applying FCMs theory into aforementioned historical accident database.
• Second, FCMs theory will be apply in order to obtain weights for those contributing factors identified from the historical accident database. These additional factors’ weightings obtained will be based on expert judgement.
• Finally, a sensitivity analysis will be performed to consolidate the results from both, historical accident data and expert opinion regarding contributing factors’ weightings.

Within MALFCM approach, each FCM is developed through establishing relationships between factors from past accident experiences and combining the results with expert opinion. Therefore, the results from the technique followed in this paper might be considered more objective, as this new approach overcomes the main disadvantage of fuzzy cognitive maps (i.e. the subjective results and knowledge deficiencies between experts).

MALFCMs method is a Fuzzy Cognitive Map-based technique, which has been designed to combine expert knowledge with lesson learnt from past accident experiences, aiming to provide results that are more reliable. As the inputs for the scenario being modelled are partially obtained from real maritime accidents, the subjective results and knowledge deficiencies between experts are reduced by applying this method. Thus, MALFCM method could be described in four main stages:

1. Historical data analysis stage.
2. Expert opinions stage
3. FCM stage
4. Consolidation of results stage

In the Historical data stage, historical data is collected for accidents with a specific profile (e.g. same vessel category involved or same navigational accident) in order to identify which contributing factors were involved in the accident case study. Then, each pair of factors is compared to create an interaction matrix, as it will be further explain later on. Furthermore, statistical analysis are performed to establish the initial state vector.

In the Expert opinion stage, experts are requested to provide their knowledge by comparing each pair of factors involved in accidents, which were identified from the historical accident database. This comparison process may be accomplished through numeric values or linguistic values. Thus, when linguistic values are applied, a conversion into fuzzy numbers is required. Finally, an interaction matrix and a state vector are created for each expert individually.

In the FCM stage, the threshold function is selected, and two FCMs are created by following Eq. (1). The first FCM is created by applying the threshold function to the interaction matrix and the state vector obtained from the Historical data stage. In addition, the second FCM integrates the interaction matrix and the state vector obtained from the expert judgement. For both FCMs created, the results are analyzed, and the obtained weightings are ranked.

(1) $$A_{i}^{\left(t+1\right)}=f\left(A_{i}^{\left(t\right)}+\sum_{j=1,\;\;j\neq i}^{n}W_{\text{ji}}A_{j}^{\left(t\right)}\right)$$

*Equation 1. Traditional formula to calculate the values of concepts in an FCM*

Lastly, in the consolidation of result stage, final weightings are obtained as a combination from the historical data and expert judgement results.

**Fig. 1:**
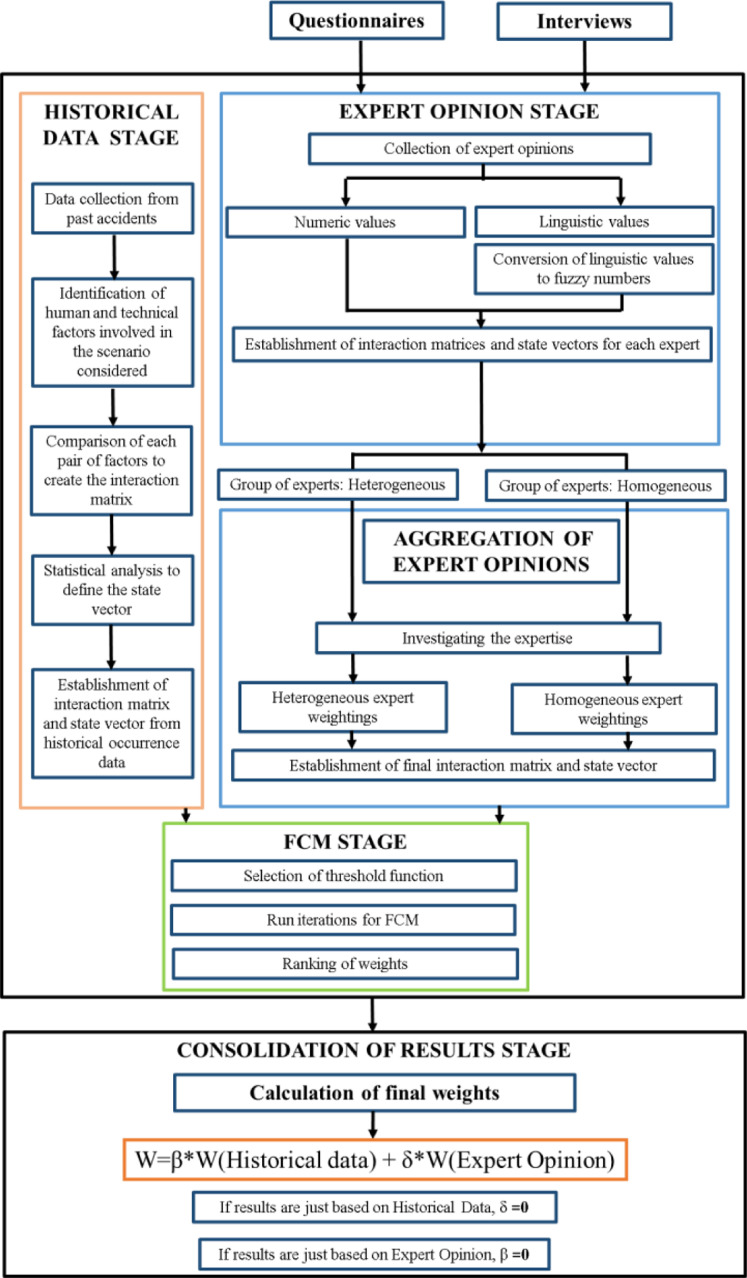
MALFCM overview.

MALFCMs model above-introduced is illustrated in Fig. 1. Furthermore, its stages are fully explained in the next sections.

## Stage 1: historical data stage

In this stage, historical occurrence data is collected for a predefined case study (e.g. a specific vessel category) in order to identify factors involved into past accidents. Once the previous factors are identified, the interaction matrix and the state vector are created. Within a traditional FCM, experts are requested to provide the strength of the relations amongst each pair of factors. However, the quality of expert's feedback depends on the experience of each expert and the relevance of his/her expertise to this topic [4]. In addition, often it is not possible to obtain reliable results due to the unavailability of relevant experts. Thus, by analyzing and considering accident historical data, it is possible to obtain more objective results, as the accidents analyzed have already taken place and therefore it is possible to track back the factors that originated each accident separately.

Henceforth, for the interaction matrix construction, each pair of factors are compared. For example, in order to determinate the relation between factors Ci and Cj, the historical accident database is filtered by the accidents caused by any of these two factors, in order to calculate how often these two factors have been recorded into past accidents. Then, the database is filtered by the accidents that register together Ci and Cj as a common accident cause. Following this process, the weight of Ci over Cj is established as the relation between the accidents with both factors in common and the accidents with Ci but not Cj. Moreover, the weight of Cj over Ci is defined as the relation between the accidents with Ci and Cj and the accidents with Cj but not Ci. Eq. (2) provides a better picture of the process being described This process is repeated in order to obtain the relations and weights of each pair of factors, creating an interaction matrix n x n, in which n shows the total number of factors being analyzed.

(2) $$W_{a,b}=\frac{W_{Fa\cap Fb}}{W_{Fa}}$$

*Equation 2. Formula to calculate the value of each component for the interaction matrix created for the historical data analysis stage.*

Moreover, the state vector is defined as the statistical occurrence of each contributing factor. Thus, for a factor Ci, its state vector value is defined as the relation of the total number of accidents with Ci involved, and the total number of accidents recorded on the historical accident database.

## Stage 2: expert opinion stage

The Expert opinion stage comprises expert participation. Through this stage, experts are requested to provide their knowledge by comparing each pair of factors Ci and Cj identified from the historical accident database, in order to complete the expert interaction matrix. This rating process might be accomplished through numeric values or linguistic terms. However, as it is extremely challenging for some expert to assign a number value in specific scenarios, an alternative solution is to apply linguistic variables. For this study, seven variables were applied as recommended by Markinos, Papageorgiou et al. [3] (i.e. very very low < very low < low < medium < high < very high < very very high). Thus, above-mentioned information is used to define an interaction matrix for each expert. Moreover, experts are asked to indicate at which level (within the interval [0,1]) a factor needs to be active in order to have a minimum contribution into an accident. This information allows defining an initial state vector for each expert.

In addition, as expertise is established with experience, some experts may be more credible than others may. Hence, it is possible to weight each expert's opinion in order to increase or reduce the importance of their feedback [1]. Therefore, when experts assigned to the case study do not have the same level of knowledge, the group is considered to be heterogeneous, and a weighting coefficient (α) is defined for each expert based on his/her knowledge. Alternatively, where all experts involved in the study are considered to have the same level of expertise, i.e. a homogeneous group, the weighting coefficient is not applied. Also, a generic interaction matrix and state vector are created for the expert group, by combining each interaction matrix and state vector through the weighting coefficient.

## Stage 3: FCM stage

In order to proceed with the FCM stage, the Logistic or Sigmoid threshold function is selected, which is shown in Eq. (3). Then, aforementioned threshold function is applied to two sets of data, creating two different FCMs. The first FCM is created by incorporating the results from the historical data stage (i.e. the interaction matrix and the state vector obtained from statistical analysis), while the second FCM integrates the findings from the expert analysis. For both FCMs, the results are analyzed separately, and the weights obtained for each contributing factor are ranked.

(3) $$f=\frac{1}{1+e^{-\lambda x}}$$

*Equation 3. Logistic signal function*

## Stage 4: consolidation of results stage

Lastly, in the consolidation of result stage, final weights for each accident-contributing factor are obtained. from historical occurrence data and expert opinion. In order to define the final factors weightings, two coefficients are established, δ and β as shown in Fig. 1. Finally, a sensitivity analysis is performed to identify the value for the two coefficients, δ and β. Thus, although MALFCM is conceptually designed to incorporate together the findings from historical data and expert opinion, it can be perfectly applied exclusively to both, historical data or expert opinion.

## Declaration of Competing Interest

The authors declare that they have no known competing financial interests or personal relationships that could have appeared to influence the work reported in this paper.
